# Genomic Features and Predicted 3D Structures of the CcWOX Transcription Factors in *Cinnamomum camphora*

**DOI:** 10.3390/ijms26178204

**Published:** 2025-08-23

**Authors:** Fengshuo Cui, Kang Wang, Haoran Qi, Tengfei Shen, Caihui Chen, Yongda Zhong, Meng Xu

**Affiliations:** 1 State Key Laboratory of Tree Genetics and Breeding, Co-Innovation Center for Sustainable Forestry in Southern China, Nanjing Forestry University, Nanjing 210037, China; cuifengshuo@njfu.edu.cn (F.C.); wk160016@outlook.com (K.W.); haoranqi@njfu.edu.cn (H.Q.); tengfeishen@njfu.edu.cn (T.S.); 2 Jiangsu Key Laboratory for Conservation and Utilization of Plant Resources, Institute of Botany, Jiangsu Province and Chinese Academy of Sciences (Nanjing Botanical Garden Mem. Sun Yat-Sen), Nanjing 210014, China; 3 Jiangxi Provincial Key Laboratory of Improved Variety Breeding and Efficient Utilization of Native Tree Species (2024SSY04092), Institute of Biological Resources, Jiangxi Academy of Sciences, Nanchang 330096, China; chencaihui0110@163.com

**Keywords:** *Cinnamomum camphora*, WUSCHEL-related homeobox, three-dimensional structures, AlphaFold3

## Abstract

The WUSCHEL-related homeobox (WOX) gene family is integral to plant growth and development. Here, we identified 14 *CcWOX* genes from the *Cinnamomum camphora* genome and analyzed their phylogeny, conserved features, and expression patterns. Phylogenetic inference grouped CcWOX into the Ancient, Intermediate, and WUS clades, consistent with other plant lineages. Expression profiling across seven tissues/organs, together with qRT-PCR validation, revealed tissue-biased expression for several members (e.g., floral or root enrichment), suggesting gene-specific roles during development. Using AlphaFold3, we predicted monomeric structures for CcWOX proteins and an interface model compatible with an interaction between CcWOX3 and CcLBD33. Consistently, bimolecular fluorescence complementation (BiFC) in *Nicotiana benthamiana* detected nuclear YFP signals for cEYFP-CcWOX3 + nEYFP-CcLBD33 relative to appropriate negative controls, confirming a physical interaction in plant cells. While these findings support a putative WOX–LBD interaction module in *C. camphora*, the regulatory functions remain to be established. Overall, this work provides a framework for dissecting the CcWOX family in *C. camphora* and illustrates how AI-assisted structure prediction can be integrated with cell-based assays to accelerate hypothesis generation in plant developmental biology.

## 1. Introduction

The development of adventitious roots represents a crucial adaptive strategy for plants to optimize growth and respond to environmental challenges. In grass species (Poaceae) such as *Oryza sativa* and *Triticum aestivum*, these specialized root structures, known as crown roots in cereals, form the primary component of the root architecture, performing essential functions in nutrient acquisition, water uptake, and mechanical stability [1,2]. Additionally, the ability to generate adventitious roots from vegetative cuttings is key for clonal propagation, ensuring genetic continuity and ecological resilience across plant populations [3].

The phytohormonal regulation of this process involves complex interactions, with auxin acting as the principal regulator. Other hormones, including cytokinin, gibberellin, and ethylene, interact with auxin to form intricate signaling networks that finely tune root development. At the genomic level, adventitious root formation is governed by the coordinated action of various signaling pathways and transcriptional regulators. Notable transcription factor families, including WUSCHEL-related homeobox (WOX) [4,5,6,7], the lateral organ boundary (LOB) domain (LBD) [8,9,10,11], and v-myb avian myeloblastosis viral oncogene homolog (MYB) [12,13], control developmental processes by reprogramming cellular differentiation pathways. WOX11 is hypothesized to play a critical role in root system architecture, as suggested by studies in *Arabidopsis* and other species. The genetic deletion of *WOX11* (*wox11* mutants) significantly impairs lateral and adventitious root proliferation while disrupting spatial patterning. In contrast, the ectopic expression of WOX11 promotes increased root branching complexity [14]. Similarly, studies show that LBD16 plays an essential, non-redundant role in root primordium initiation, with lbd16 mutants failing to form adventitious roots in excised leaf tissues [8]. Mechanistically, it has been suggested that WOX11 works in concert with LBD16 through both genetic epistasis and direct protein interactions to enhance root development [15].

Studies across various species have confirmed the evolutionary conservation of the WOX11-LBD16 regulatory module in adventitious root formation. In rice, for example, WOX11 directly binds to the promoter region of LBD16, recruiting the histone demethylase JMJ706 to remove H3K9me2 modifications at the LBD16 locus, thereby activating its transcription. This mechanism precisely regulates crown root formation and growth. Additionally, it has been suggested that LBD16 inhibits its overexpression through a feedback loop, wherein accumulated LBD16 protein competes with WOX11, disrupting the WOX11-JMJ706 complex and reducing transcriptional activation of LBD16. This feedback loop maintains a dynamic balance in *LBD16* expression, preventing detrimental effects on plant development [16]. In *A. thaliana*, WOX11 has been shown to regulate dedifferentiation and redifferentiation processes by activating LBD16, thereby maintaining gene expression homeostasis. Similar functional conservation has been observed in woody plants such as *Populus tomentosa*, where overexpression of WOX11 promotes adventitious root formation, while knockdown impairs root development [17]. Synergistic interactions with other LBD family members further reinforce the evolutionary conservation of the WOX11-LBD16 module in adventitious root development. This conserved molecular module may aid in understanding the universal mechanisms of adventitious root formation but also serves as an important reference for studying similar modules in other plant species [14,18].

The WUSCHEL-related homeobox (WOX) gene family is a specialized subgroup within the homeobox superfamily, encoding transcription factors characterized by a conserved helix–loop–helix–turn–helix homeodomain (HD) of approximately 60 residues [19]. A distinctive feature of WOX proteins is the unique interhelical insertions between helices 1–2 and 2–3, a trait not found in other homeodomain proteins [20]. Phylogenetic analysis based on sequence homology divides WOX proteins into three evolutionary clades: the functionally specialized WUS clade, the ancestral Ancient clade, and the transitional Intermediate clade [21]. Members of the WUS clade contain a signature WUS-box motif (T-L-X-L-F-P-X-X) in addition to their HD architecture, while sequence divergence in this region is observed in the Ancient and Intermediate clades. As plant-exclusive transcriptional regulators, WOX proteins control various developmental processes, from embryogenesis to stem cell niche maintenance [22,23,24], and have well-documented roles in organogenesis, including adventitious/lateral root formation [25,26], ovules [27], and floral morphogenesis [28,29,30].

The archetypal WUSCHEL (WUS) protein, first characterized in *Arabidopsis thaliana* [31], exemplifies the regulatory breadth and developmental importance of the WOX family. WUS is specifically expressed in the organizing center (OC) of the shoot apical meristem (SAM), where it acts as a master regulator of stem cell maintenance by repressing differentiation signals through a non-cell-autonomous mechanism. It functions via a negative feedback loop with the CLAVATA (CLV) signaling pathway to precisely balance stem cell proliferation and differentiation [32]. Loss-of-function wus mutants exhibit premature stem cell exhaustion and termination of meristem activity, while ectopic WUS expression induces callus-like overproliferation of undifferentiated cells [31]. The *A. thaliana* genome encodes 15 AtWOX isoforms, which collectively orchestrate key developmental programs, including embryonic patterning, stem cell homeostasis, and organogenesis [21,31,33]. For example, AtWOX2 specifies apical cell fate during zygotic development [33], while AtWOX5 maintains the quiescent center identity in root apical meristems [34], and AtWOX6 further regulates ovule differentiation [27]. Beyond herbaceous species, WOX gene function is conserved across woody plants: Transgenic overexpression of *PtoWOX5a* in *Populus tomentosa* disrupts root architecture, leading to hyperproliferation of adventitious roots, accompanied by suppressed primary root elongation [35]. In hybrid poplar (*P. deltoides* × *P. euramericana* ‘Nanlin895′), ectopic expression of *PeWOX11a/b* induces ectopic root primordia formation on cuttings and aerial tissues, while coordinating the development of root–axillary bud–leaf developmental axes [36]. Studies in conifers reveal that PaWOX2 suppression impedes embryogenesis, preventing proto-epithelial tissue differentiation [37]. Taken together, WOX proteins act as master regulators of meristematic activity, ensuring stem cell maintenance and developmental priming in both root and shoot apical meristems of woody plants [21,31,38].

*Cinnamomum camphora* (camphor tree), a prominent species in the subtropical evergreen broad-leaved forests of East Asia, is valued for its terpene compounds and holds significant economic, cultural, and ecological importance [39,40]. While WOX transcription factors have been identified in other woody plants such as *P. trichocarpa* [41], *Eriobotrya japonica* [42], and *Citrus reticulata* [43], no studies have yet characterized these genes in *C. camphora*. Utilizing our previously sequenced high-quality *C. camphora* genome [44], we systematically identified and analyzed the *CcWOX* genes for the first time. Additionally, we investigated the role of the conserved WOX11-LBD16 module in adventitious root development in *C. camphora*. Using the AI-based program AlphaFold3, we predicted the three-dimensional structures of CcWOX proteins and analyzed the potential interaction between CcWOX3 and CcLBD33. These interactions were predicted and are being further validated experimentally through bimolecular fluorescence complementation (BiFC) assays. While the current study provides valuable predictions and insights, functional validation, particularly regarding adventitious root formation and reproductive development, will be a key focus of our future research. Our findings provide valuable insights into the potential roles of WOX transcription factors in woody plants and offer a foundation for future functional studies.

## 2. Results

### 2.1. Identification of WOX Genes in C. camphora

To identify *WOX* genes in *C. camphora*, we employed a combination of Hidden Markov Model (HMM) searches and BLASTp analysis targeting the homeodomain (HD). Cross-referencing the results from these two methods confirmed the presence of the HD in the candidate proteins, leading to the identification of 14 *CcWOX* genes. These genes were designated *CcWOX1* to *CcWOX14* based on their chromosomal positions (Table 1). The predicted protein lengths of the *CcWOX* genes range from 167 amino acids (CcWOX10) to 524 amino acids (CcWOX12). Most of these proteins have molecular weights between 20 and 30 kDa, except for CcWOX12, which has a molecular weight of 56.83 kDa. The theoretical isoelectric points (pI) of the proteins vary from 5.11 (CcWOX1) to 9.54 (CcWOX5), with instability indices ranging from 41.86 to 81.23. Additionally, the negative GRAVY (grand average of hydropathicity) values suggest that all CcWOX proteins are hydrophilic. Subcellular localization predictions indicate that all CcWOX proteins are predominantly localized in the nucleus. Notably, CcWOX12 is predicted to localize to the cytoplasm, suggesting potential unique functional roles for this protein compared to other members of the WOX family.

### 2.2. Phylogenetic and Conserved Domain Analysis

**Figure 1:**
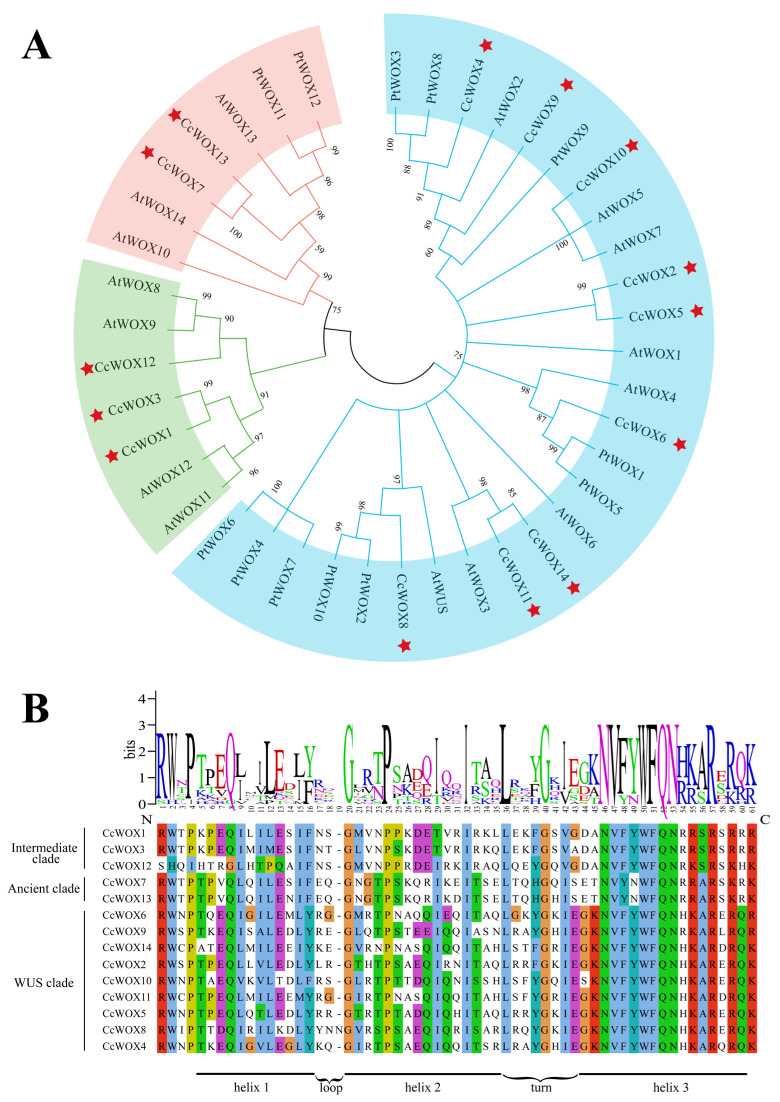
Evolutionary divergence and structural conservation of CcWOX homeodomains. (**A**) Maximum-likelihood phylogeny of WOX orthologs from *C. camphora* (CcWOX), *P. trichocarpa* (PtWOX), and *A. thaliana* (AtWOX). The phylogenetic tree delineates three evolutionary lineages: the Ancient clade (red), the Intermediate clade (green), and the WUS clade (blue). Red stars indicate the WOX proteins identified from *C. camphora* in this study. (**B**) Structural conservation of the homeodomain (HD) across CcWOX proteins. Multiple sequence alignment reveals a 61-residue HD with a conserved helix–loop–helix–turn–helix architecture (highlighted in yellow), critical for nuclear localization and DNA target recognition.

**Figure 2:**
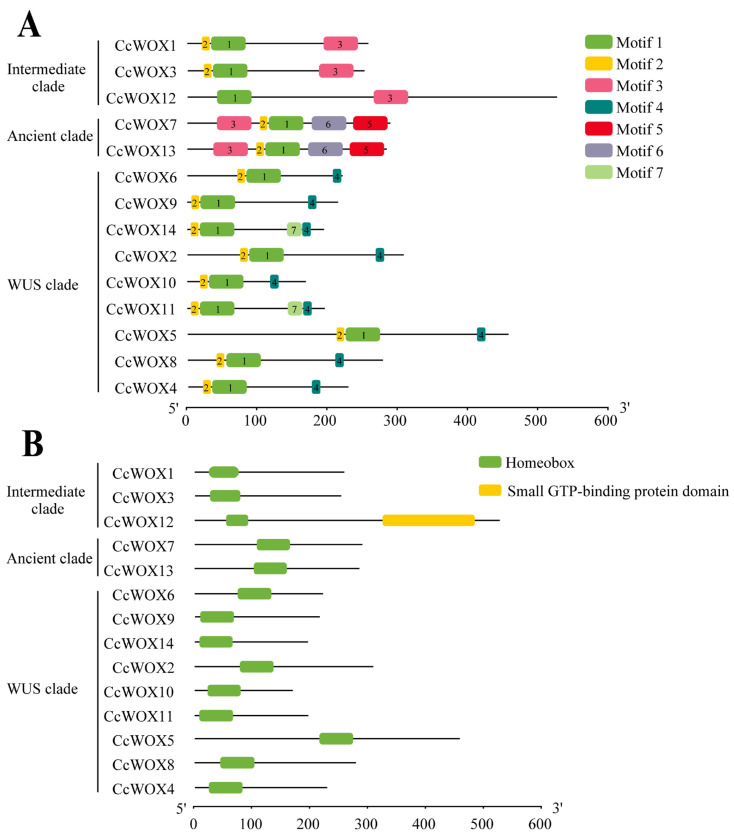
Modular organization of conserved motifs and functional domains in CcWOX proteins. (**A**) MEME-based motif profiling identifies clade-specific signatures. (**B**) Domain architecture analysis reveals atypical structural features. CcWOX12 uniquely contains a C-terminal GTPase-like domain, suggesting potential roles in signal transduction beyond canonical transcriptional regulation.

Comprehensive phylogenetic reconstruction revealed evolutionary trajectories among *C. camphora* (CcWOX), *A. thaliana* (AtWOX), and *P. trichocarpa* (PtWOX) transcription factors (Figure 1A). Hierarchical clustering categorized CcWOX members into three phylogenetically distinct lineages: the ancestral Ancient clade (CcWOX7, CcWOX13), the transitional Intermediate clade (CcWOX1, CcWOX3, CcWOX12), and the evolutionarily dynamic WUS clade (CcWOX2, CcWOX4–6, CcWOX8–11, CcWOX14). Notably, the WUS clade exhibited significant gene expansion, which could imply clade-specific selective pressures and functional specialization during evolution. Conservation analysis through multiple sequence alignment identified a signature N-terminal homeodomain (HD) spanning 61 residues in all CcWOX proteins, featuring the characteristic helix–loop–helix–turn–helix topology critical for nuclear targeting and DNA interaction (Figure 1B) [45]. Cladistic divergence was evident through variable sequence architectures in non-HD regions, particularly within the Intermediate and Ancient clades. Clade-specific variations in the HD were observed, with specific residues at positions 43, 45, and 60 distinguishing the WUS clade from the Ancient and Intermediate clades. Conserved motif analysis using MEME identified seven distinct motifs in CcWOX proteins (Figure 2A). Motifs 1 and 2 are part of the HD and are present in all proteins, whereas motifs 5 and 6 are unique to the Ancient clade, and motif 3 is specific to the Intermediate clade. Notably, motif 4, corresponding to the WUS-box domain (E-T-L-E-L-F-P-L-R-S-T-G-I), is exclusively found in the WUS clade. This domain is associated with stem cell regulation and floral tissue development. Furthermore, CcWOX12 harbors a small GTP-binding domain (amino acids 323–482) near its C-terminal region (Figure 2B), which may be associated with its cytoplasmic localization and possible involvement in vesicle transport. [46].

### 2.3. Synteny Analysis

**Figure 3:**
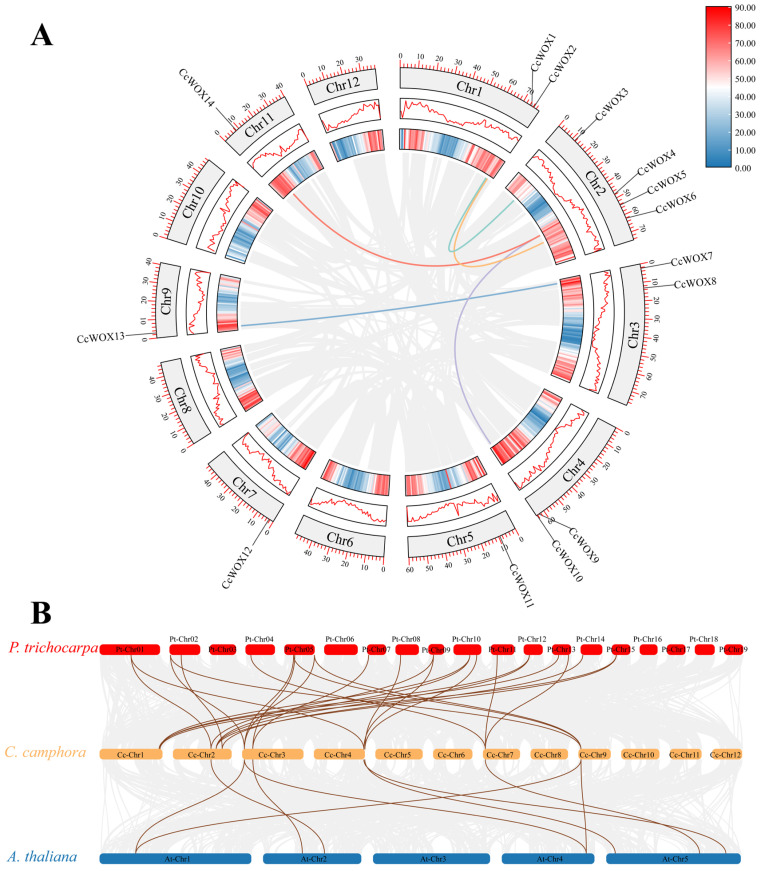
Synteny analysis of *CcWOX* genes. (**A**) Syntenic relationships of duplicated *CcWOX* genes in the *C. camphora* genome, with colored lines connecting paralogous gene pairs. (**B**) Comparative synteny analysis of the *C. camphora*, *P. trichocarpa*, and *A. thaliana* genomes. Brown lines indicate syntenic *WOX* genes pairs, suggesting evolutionary conservation and gene duplication events.

Gene duplication mechanisms, including segmental and tandem duplications, are key drivers of gene family diversification in plant genomes [47,48]. To elucidate expansion dynamics within the CcWOX family, we performed comparative synteny mapping across species (Figure 3). Intergenomic collinearity analysis identified 26 orthologous *WOX* gene pairs between *C. camphora* and *P. trichocarpa*, and 9 orthologs shared with *A. thaliana* (Figure 3B), suggesting ancient conservation of *WOX* genes predating speciation events. Intragenomic duplication analysis revealed five paralogous *CcWOX* clusters: *CcWOX1-CcWOX3*, *CcWOX2-CcWOX5, CcWOX4-CcWOX14, CcWOX4-CcWOX9,* and *CcWOX7-CcWOX13*. Chromosomal localization of these paralogs strongly supports segmental duplication as the dominant evolutionary mechanism, with syntenic blocks distributed across nonhomologous chromosomes. This genomic architecture further supports paleo-polyploidization events in *C. camphora*’s evolutionary history [49].

### 2.4. Expression Patterns of CcWOX Genes in Different Tissues

**Figure 4:**
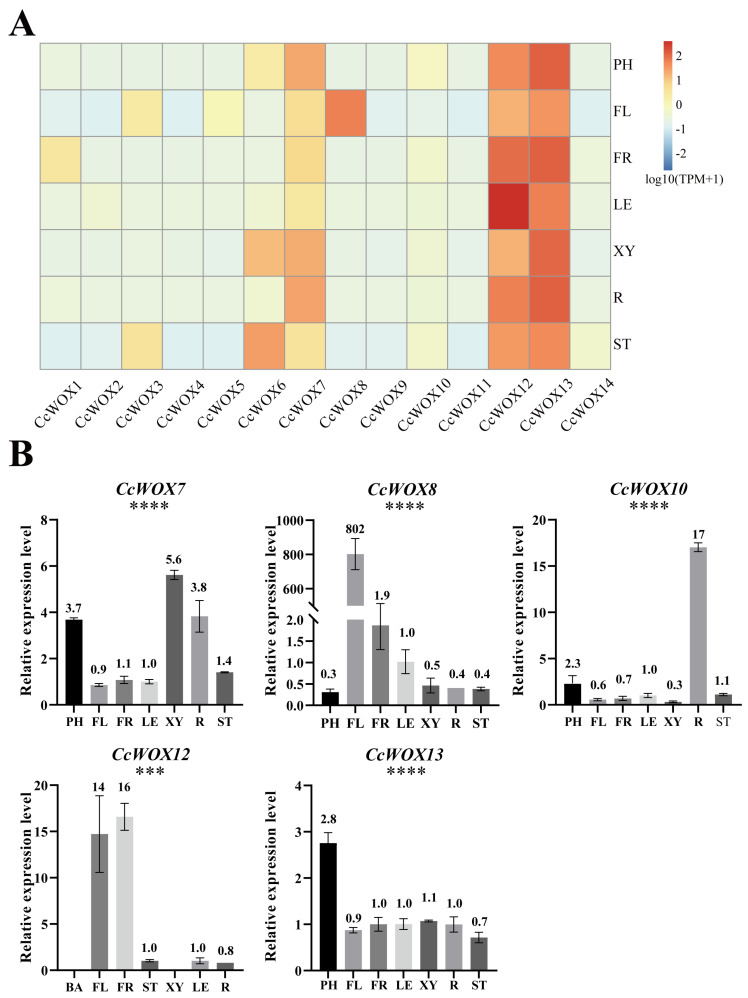
Heatmap and qRT-PCR validation of *CcWOX* gene expression patterns across seven tissues/organs. (**A**) Heatmap illustrating the expression levels of 14 *CcWOX* genes across seven tissues/organs: trunk phloem (PH), flowers (FL), fruits (FR), leaves (LE), developing xylem (XY), roots (R), and young stems (ST). Low expression levels are represented in blue, while high expression levels are indicated in red. (**B**) qRT-PCR validation of five selected *CcWOX* genes across the same tissues/organs. All experiments were performed with three biological replicates, and error bars represent the standard deviation. Raw datapoints are overlaid on each bar to illustrate replicate variation. Statistical significance was determined by one-way ANOVA followed by Tukey’s HSD post hoc test across all tissues/organs. Asterisks indicate that the corresponding gene shows significant expression differences across all tissues/organs (* *p* < 0.05, ** *p* < 0.01, *** *p* < 0.001, **** *p* < 0.0001).

Previous studies have shown that *AtWOX* genes exhibit tissue-specific expression patterns. To investigate the expression patterns of *CcWOX* genes, we analyzed RNA-seq data from seven tissues/organs of *C. camphora*: flowers, leaves, fruits, roots, young stems, developing xylem, and trunk phloem. Gene expression levels were measured in transcripts per million (TPM). The results revealed generally low expression levels for *CcWOX* genes across the analyzed tissues, with notable variation in specific tissues (Figure 4A). For example, *CcWOX6* showed higher expression in young stems and developing xylem, while *CcWOX8* exhibited elevated expression in flowers, suggesting its potential involvement in floral organ development. Interestingly, CcWOX12 and CcWOX13 demonstrated consistently higher expression levels across all tissues (TPM > 20), suggesting their potentially critical roles throughout the developmental stages of *C. camphora*. To validate the RNA-seq results, qRT-PCR experiments were conducted for selected *CcWOX* genes across the same seven tissues/organs. The qRT-PCR results confirmed the tissue-specific expression patterns (Figure 4B). For instance, *CcWOX7* showed high expression in developing xylem, trunk phloem, and roots; *CcWOX10* exhibited high expression specifically in roots; *CcWOX12* displayed elevated expression in flowers and fruits; and *CcWOX13* was predominantly expressed in trunk phloem. Notably, *CcWOX8* showed extremely higher expression in flowers, strongly suggesting its significant role in reproductive development, consistent with findings in other plant species [50,51]. Statistical significance analysis was performed using one-way ANOVA, and the results showed significant differences (*p* < 0.05) in the expression levels between different tissues for certain genes, confirming the observed tissue-specific expression patterns.

### 2.5. CcWOX Three-Dimensional Structure and Interaction Prediction

**Figure 5:**
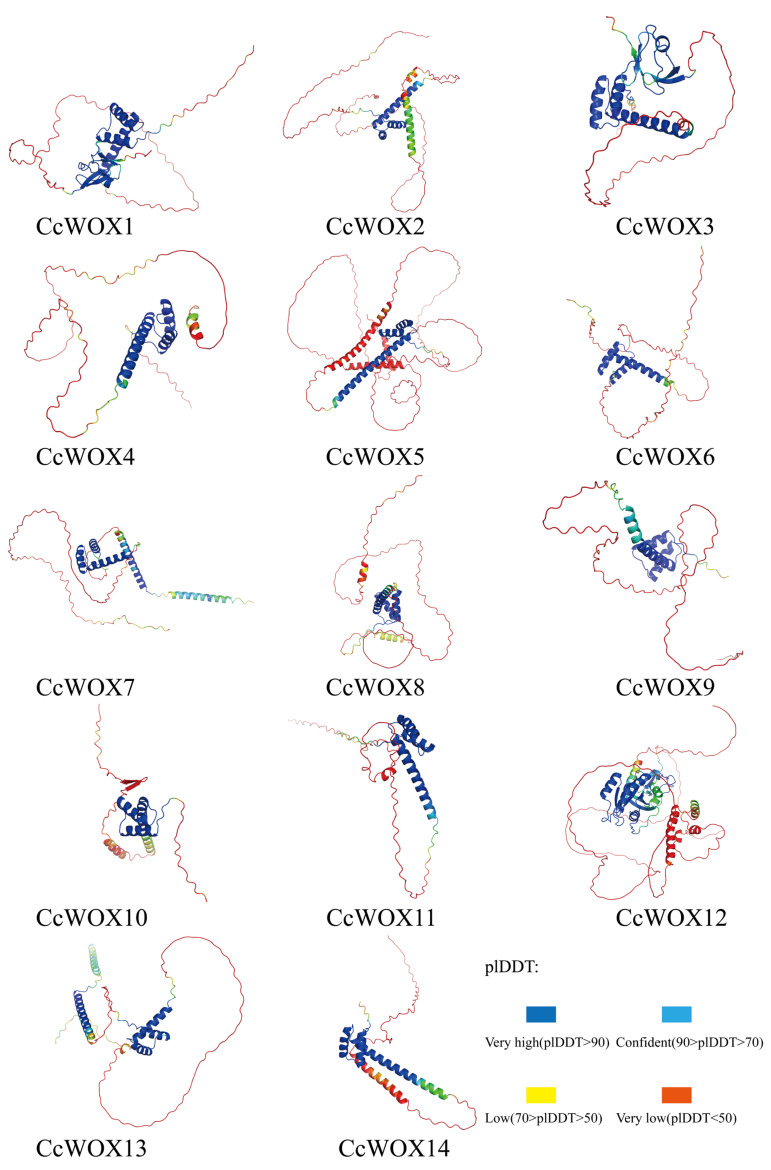
Three-dimensional structure of CcWOX protein predicted de novo using AlphaFold3. Structure is color-coded according to pLDDT values, with higher scores indicating greater confidence.

**Figure 6:**
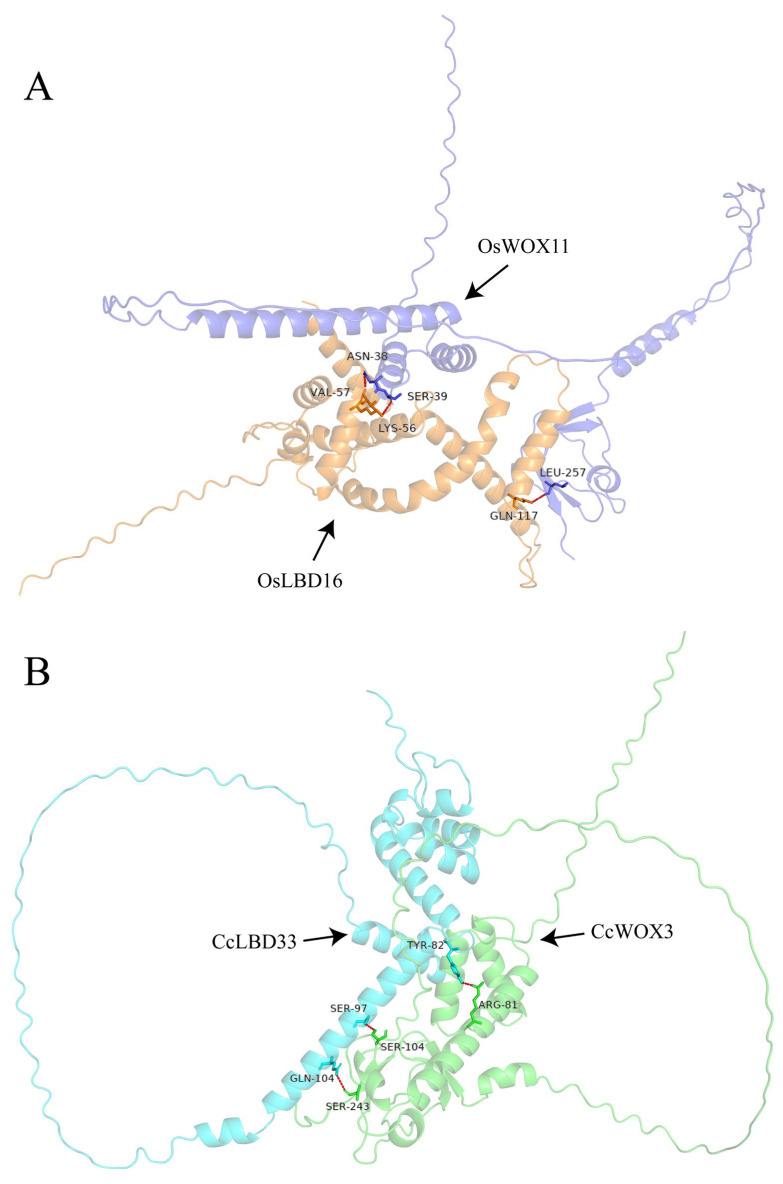
Interaction models of WOX and LBD protein complexes in *O. sativa* and *C. camphora*. (**A**) Interaction model between OsWOX11 and OsLBD16 in *O. sativa*, showing interacting residues (ASN-38, SER-39, LEU-257) and hydrogen bonds (red dashed lines). (**B**) Interaction model between CcWOX3 and CcLBD33 in *C. camphora*, showing interacting residues (ARG-81, SER-104, SER-243) and hydrogen bonds (red dashed lines).

Using AlphaFold3, we performed de novo predictions of the three-dimensional structures of CcWOX transcription factors. The model with the highest confidence for each protein was selected for visualization (Figure 5). While variations were observed in the overall conformations of the predicted structures, all models consistently identified the conserved helix–loop–helix–turn–helix homeodomain (HD) structure. Predicted local-distance difference test (pLDDT) scores, ranging from 0 to 100, were used to assess the confidence of each amino acid’s structural prediction, with higher scores indicating greater reliability. Notably, the conserved HD structure exhibited consistently high pLDDT scores, reflecting AlphaFold3’s confidence in its structural accuracy. However, it is important to note that these predictions are computational and should be experimentally validated. Sequence alignment revealed homologous proteins in *C. camphora* corresponding to the rice (Oryza sativa) transcription factors WOX11 and LBD16, designated as CcWOX3 and CcLBD33, respectively. AlphaFold3 was further used to predict interactions between these proteins [52,53]. Among the generated models, the most reliable cluster was selected for detailed visualization using PyMOL, highlighting hydrogen bonds and interacting amino acid residues within the protein complex (Figure 6). The results revealed three hydrogen bonds distributed from the N-terminus to the C-terminus of the CcWOX3-CcLBD33 complex. Specifically, three residues in CcWOX3-ARG-81, SER-104, and SER-243—formed hydrogen bonds with CcLBD33. Similarly, the rice OsWOX11-OsLBD16 complex exhibited three hydrogen bonds formed by residues ASN-38, SER-39, and LEU-257 in OsWOX11. Interestingly, no hydrogen bonds were observed in the DNA-binding domain of CcWOX3 (amino acid positions 28–81). These findings suggest that the interaction occurs at the protein-protein level, independent of DNA-binding functions, pending further validation. However, this interaction does not confirm a functional regulatory relationship, as the experimental validation is still pending. It is important to clarify that while these computational results provide valuable insights, further experimental evidence is necessary to confirm the biological significance of the interaction between CcWOX3 and CcLBD33. The potential regulatory role of this interaction in processes like adventitious root formation remains speculative, and further research is required to explore the functional implications of this interaction.

### 2.6. Validation of CcWOX3 and CcLBD33 Interaction Using BiFC Assay

To validate the interaction between CcWOX3 and CcLBD33 proteins in vivo, bimolecular fluorescence complementation (BiFC) assays were performed in Nicotiana benthamiana epidermal cells. The full-length coding sequences (CDS) of CcLBD33 and CcWOX3 were fused to the N-terminal (nEYFP) and C-terminal (cEYFP) fragments of enhanced yellow fluorescent protein (EYFP), respectively, generating the constructs nEYFP-CcLBD33 and cEYFP-CcWOX3. These constructs were transiently co-expressed in N. benthamiana cells via agroinfiltration, and fluorescence signals were detected using a confocal laser scanning microscope.

**Figure 7:**
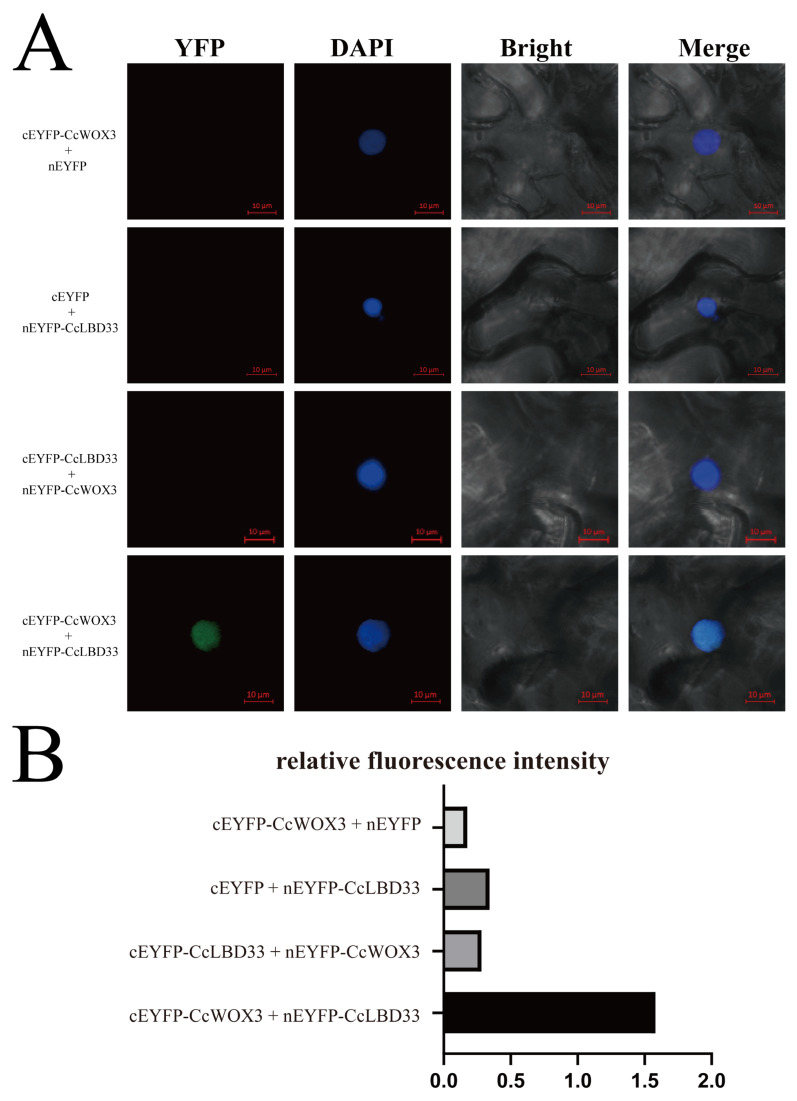
BiFC analysis of CcWOX3 and CcLBD33 interactions in Nicotiana benthamiana epidermal cells. (**A**) Representative BiFC images showing YFP fluorescence signals. Strong YFP signals were observed in cells co-expressing cEYFP-CcWOX3 and nEYFP-CcLBD33, confirming the interaction between the two proteins. No fluorescence was detected in the negative controls (cEYFP-CcWOX3 + nEYFP and cEYFP + nEYFP-CcLBD33) or in the swapped constructs (nEYFP-CcWOX3 + cEYFP-CcLBD33). The “YFP” panel shows fluorescence signals, the “DAPI” panel displays nuclear staining, the “Bright” panel shows bright-field images, and the “Merge” panel overlays the three channels. Scale bars: 10 µm. (**B**) Quantitative analysis of YFP fluorescence intensity. Fluorescence intensity was measured using ImageJ (v1.54g) by defining nuclear regions (based on the DAPI channel) and quantifying the mean intensity of the YFP channel. Each nucleus was treated as an independent datapoint, and normalized values were plotted in GraphPad Prism (v10.4.0). Error bars represent the standard deviation of biological replicates, and individual datapoints are shown. The experimental group (cEYFP-CcWOX3 + nEYFP-CcLBD33) exhibited significantly higher fluorescence compared with the negative controls.

As shown in Figure 7A, strong YFP fluorescence signals were observed in cells co-expressing cEYFP-CcWOX3 and nEYFP-CcLBD33, confirming successful EYFP reconstitution and a specific interaction between the two proteins, consistent with the predicted protein–protein interaction. In contrast, no fluorescence signals were detected in the negative controls, including cEYFP-CcWOX3 + nEYFP and cEYFP + nEYFP-CcLBD33. We also tested the swapped constructs (nEYFP-CcWOX3 + cEYFP-CcLBD33), which failed to produce fluorescence. We attribute this negative result to possible steric hindrance or spatial orientation effects that prevented EYFP reassembly, reflecting a known limitation of BiFC assays rather than the absence of protein interaction. To quantitatively assess the BiFC results (Figure 7B), YFP images were processed using ImageJ (v1.54g). After channel separation, nuclear regions were defined based on the DAPI channel, and mean fluorescence intensity was measured within each nucleus in the YFP channel. Each nucleus was treated as an independent datapoint, and the data were normalized and analyzed using GraphPad Prism (v10.4.0). The quantification confirmed that the experimental group (cEYFP-CcWOX3 + nEYFP-CcLBD33) displayed significantly higher fluorescence intensity compared with the negative controls, supporting a specific in planta interaction between *CcWOX3* and *CcLBD33*.

Together, these results validate the interaction between CcWOX3 and CcLBD33 in plant cells, corroborating the bioinformatics-based interaction predictions and their nuclear subcellular localization. We also acknowledge the technical limitations of BiFC and note that additional assays such as co-immunoprecipitation or pull-down will be required in future work to further substantiate these findings.

## 3. Discussion

In this study, we identified 14 *CcWOX* genes in the genome of *C. camphora*, which were classified into three evolutionary clades: Ancient, Intermediate, and WUS. This classification is consistent with the evolutionary organization of *WOX* genes observed in model plants such as *Arabidopsis thaliana* and *P. trichocarpa* [21]. The homeodomain (HD) characteristic of the Homeobox superfamily was highly conserved across *CcWOX* proteins [20]. Notably, a 61–amino acid HD was observed in several members rather than the canonical 60 aa; this difference likely reflects lineage-specific divergence and may contribute to subfunctionalization rather than indicating major functional shifts. The sharing of conserved motifs within clades points to a degree of functional conservation, whereas five paralogous gene pairs suggest that duplication has contributed to the expansion and diversification of the *CcWOX* family in *C. camphora*.

Beyond evolutionary features, our expression analyses revealed clear tissue/organ-biased patterns for several *CcWOX* genes. RNA-seq heatmaps together with qRT-PCR validation (one-way ANOVA followed by Tukey’s HSD; raw datapoints overlaid) indicated that, for example, *CcWOX8* is elevated in flowers and *CcWOX10* is enriched in roots, while *CcWOX12* and *CcWOX13* showed broadly higher baseline expression across multiple tissues/organs. These observations suggest potential roles for *CcWOX* genes in reproductive and vascular development, consistent with reports from other plant species, while also underscoring gene-specific differences within the family [54,55].

Given the conservation of WOX–LBD modules in other plants (e.g., WOX11–LBD16 in crown/axillary root programs), we explored whether a similar regulatory architecture might exist in *C. camphora*. Using AlphaFold3, we generated monomeric structures of CcWOX3 and CcLBD33 and built multimeric interface models; the predicted interface topology was compatible with a potential interaction. Consistent with these predictions, BiFC assays in *N. benthamiana* epidermal cells detected nuclear YFP signals when co-expressing cEYFP-CcWOX3 with nEYFP-CcLBD33. Crucially, both categories of negative controls (cEYFP-CcWOX3 + nEYFP and cEYFP + nEYFP-CcLBD33) were dark, and the swapped constructs (nEYFP-CcWOX3 + cEYFP-CcLBD33) did not fluoresce—likely due to orientation/steric constraints inherent to BiFC rather than a lack of interaction. Quantification was performed at the level of individual nuclei (each nucleus treated as one datapoint) and confirmed significantly higher fluorescence in the experimental combination relative to controls. Together, these results confirm an in planta physical interaction between CcWOX3 and CcLBD33 and support the existence of a WOX–LBD module in *C. camphora*.

We note several limitations and future directions. First, while the interface predicted by AlphaFold3 is consistent with the BiFC result, computational modeling provides hypotheses rather than definitive binding energetics. Second, BiFC can be sensitive to fusion orientation and may yield false negatives in swapped constructs; therefore, complementary assays (e.g., co-immunoprecipitation, pull-down, or yeast two-hybrid) and genetic tests (overexpression/knockdown) will be valuable to define the regulatory hierarchy and developmental contexts in which CcWOX3 and CcLBD33 act.

In conclusion, our work provides a comprehensive view of the CcWOX family, integrates expression profiling with AlphaFold3-guided structural modeling, and delivers experimental evidence for a nuclear CcWOX3–CcLBD33 interaction in plant cells. These findings establish a foundation for dissecting WOX-mediated developmental programs in *C. camphora* and illustrate how AI-assisted structure prediction can be combined with cell-based assays to accelerate hypothesis testing in plant molecular biology.

## 4. Materials and Methods

### 4.1. Plant Materials

The experimental material consisted of three-year-old clonal propagules of *C. camphora* cultivar ‘Gantong 1’. Seven distinct tissues/organs, including reproductive structures (fruits, flowers), vegetative organs (leaves, roots, young stems), and specialized tissues/organs (developing xylem, trunk phloem), were collected. All specimens were rapidly cryopreserved in liquid nitrogen immediately post-harvest and subsequently stored at −80 °C to maintain biomolecular integrity. Triplicate biological replicates were systematically included in all experimental procedures to ensure methodological rigor and data reproducibility.

To isolate developing xylem and trunk phloem, sterile surgical blades were used to carefully separate tissues from the cleaned tree trunks, ensuring that only the desired tissues were collected. Trunk phloem was collected by scraping the soft inner bark (phloem layer), while developing xylem was obtained by scraping the region just inside the vascular cambium, targeting newly formed xylem and avoiding mature heartwood. All specimens were rapidly cryopreserved in liquid nitrogen immediately post-harvest, followed by storage at −80 °C to maintain biomolecular integrity until further processing.

### 4.2. Bioinformatics Analysis

Genomic resources for *C. camphora* (GWHBGBX00000000) were obtained from the Genome Warehouse database [44]. A dual-strategy identification pipeline was established for *CcWOX* gene discovery, which combined both BLASTp homology search and HMM profiling. First, canonical AtWOX protein sequences from TAIR (https://www.arabidopsis.org, accessed on 25 November 2023) were used as queries for BLASTp homology searches with stringent criteria: sequence identity >50%, alignment length >50%, and e-value < 1 × 10^−5^, ensuring reliable sequence alignments. Concurrent with the BLASTp approach, the WOX domain HMM profile (PF00046) from Pfam [56] was employed in TBtools (v2.225) HMM searches, applying equivalent stringency thresholds to enhance gene identification accuracy [57]. The results from both approaches were cross-referenced to identify consensus candidates, which were further verified through InterPro domain analysis (https://www.ebi.ac.uk/interpro, accessed on 11 December 2023) to confirm the presence of the characteristic HD domain. Protein characterization included: Biophysical profiling via ProtParam (https://web.expasy.org/protparam/, accessed on 30 December 2023) to determine molecular weight, instability indices, and hydrophobicity [58]; Subcellular localization predictions using Plant-mPLoc (http://www.csbio.sjtu.edu.cn/bioinf/plant-multi/, accessed on 12 January 2024) [59]. Comparative phylogenomics was conducted by: Curating WOX orthologs from *A. thaliana* (TAIR) and *P. trichocarpa* (Phytozome v13) [60]; Visualizing evolutionary relationships using FigTree (v1.4.4)-enhanced dendrograms. Synteny relationships were determined through MCScanX-based collinearity detection integrated in TBtools (v2.225) [61]. All sequence alignments and annotations were visualized using Jalview (v2.11.4.1) and Tbtools graphical interfaces.

### 4.3. Quantitative Expression Analysis

RNA-seq transcriptome data were used to analyze the expression of *CcWOX* genes across various *C. camphora* tissues/organs, including reproductive structures (fruits, flowers), vegetative organs (leaves, roots, young stems), and specialized tissues/organs (developing xylem and trunk phloem). Expression levels were visualized using TBtools (v2.225).

To validate the RNA-seq data, total RNA was extracted using the RNAprep Pure Plant Plus Kit (Tiangen Biologicals, Beijing, China). RNA integrity was assessed via 1% agarose gel electrophoresis to ensure high-quality RNA for subsequent analysis. Reverse transcription was carried out using TaKaRa’s 5× PrimeScript RT Master Mix. Quantitative primers for *CcWOX* genes were designed using BeaconDesigner 8 (v8.21) software. Real-time quantitative PCR (qRT-PCR) was performed using PowerUp™ SYBR™ Green Master Mix (Takara Bio USA, San Jose, CA, USA) and analyzed on the Applied Biosystems ViiA™ 7 system(Applied Biosystems, Waltham, MA, USA). CcActin was used as the reference gene for normalization. Relative expression levels were calculated using the 2–ΔΔCt method. Statistical analysis was conducted using one-way ANOVA followed by Tukey’s HSD post hoc test to evaluate pairwise differences across all tissues/organs. Significance thresholds were set at *p* < 0.05. Raw datapoints from biological replicates were included in the visualization to illustrate data distribution and variability.

### 4.4. Three-Dimensional Structures and Interaction Prediction

The three-dimensional structures of CcWOX proteins were predicted using AlphaFold3 (DeepMind and Isomorphic Labs, https://alphafoldserver.com/, accessed on 12 June 2024) based on their amino acid sequences, providing structural insights into their potential functions. Molecular docking simulations between CcWOX3 and CcLBD33 were performed using AlphaFold3, allowing us to predict the interaction model. The predicted structural models were downloaded and visualized using PyMOL to identify key interacting amino acid residues.

### 4.5. Bimolecular Fluorescence Complementation (BiFC)

The BiFC assay was conducted to examine the in vivo interaction between CcWOX3 and CcLBD33 proteins. The N-terminal (nEYFP, residues 1–173) and C-terminal (cEYFP, residues 174–237) fragments of enhanced yellow fluorescent protein (EYFP) were PCR-amplified and cloned into the Trans-T1 vector, generating the base constructs Trans-nEYFP and Trans-cEYFP. The full-length coding sequences of CcLBD33 and CcWOX3 were fused in-frame to nEYFP and cEYFP, respectively, resulting in the chimeric constructs Trans-nEYFP::CcLBD33 and Trans-cEYFP::CcWOX3.

All constructs were confirmed by Sanger sequencing before transformation into *Agrobacterium tumefaciens* GV3101. For transient expression, bacterial suspensions were co-infiltrated into *Nicotiana benthamiana* leaves using standard protocols [62]. A total of four BiFC combinations were tested: (1) cEYFP-CcWOX3 + nEYFP-CcLBD33 (experimental group), (2) cEYFP-CcWOX3 + nEYFP (negative control), (3) cEYFP + nEYFP-CcLBD33 (negative control), and (4) cEYFP-CcLBD33 + nEYFP-CcWOX3 (swapped constructs). Infiltrated leaves were incubated in darkness at 22 °C for 48 h. Fluorescence signals were imaged using a Zeiss LSM 710 confocal laser scanning microscope. YFP excitation was performed at 514 nm, and emission was collected between 519 and 620 nm. DAPI (4′,6-diamidino-2-phenylindole) staining was used to label nuclei and assess subcellular localization.

For quantification, fluorescence images were processed using ImageJ (v1.54g). YFP and DAPI channels were separated, and nuclear regions were manually defined based on DAPI signals. Mean fluorescence intensity within each nucleus (nucleus = one datapoint) was measured from the YFP channel. Fluorescence values were normalized and plotted using GraphPad Prism 10 (v10.4.0).

## 5. Conclusions

In this study, we identified 14 CcWOX genes in the *Cinnamomum camphora* genome and characterized their phylogenetic placement, gene/protein features, and predicted three-dimensional structures. Expression profiling together with qRT-PCR validation (ANOVA with Post Hoc testing) revealed tissue/organ-biased patterns for several members, suggesting gene-specific roles in developmental processes. Using AlphaFold3, we obtained structural models for CcWOX proteins and a multimeric interface model compatible with an interaction between CcWOX3 and CcLBD33. Consistently, BiFC assays in plant cells detected a nuclear physical interaction between these two proteins relative to appropriate negative controls. These results provide a framework for dissecting WOX-mediated programs in *C. camphora* and illustrate how AI-assisted structure prediction can be integrated with cell-based assays to accelerate hypothesis generation in plant molecular biology. We note that the interaction and its regulatory consequences will require orthogonal validation (e.g., co-immunoprecipitation/pull-down/yeast two-hybrid) and genetic tests (loss- and gain-of-function) in future work.

## Figures and Tables

**Table 1:** Detailed molecular characteristics of CcWOX proteins.

Gene ID	Name	Protein Lengths (aa)	Molecular Weight (kDa)	Isoelectric Points (pI)	Instability Indices	Hydrophilicity	Predicted Subcellular Locations
Ccam01T003400	CcWOX1	256	27.89	5.11	81.23	−0.251	Nucleus
Ccam01T003479	CcWOX2	306	34.97	6.27	73.33	−0.709	Nucleus
Ccam02T000641	CcWOX3	250	27.75	5.65	73.29	−0.429	Nucleus
Ccam02T001857	CcWOX4	226	25.47	6.71	70.43	−0.781	Nucleus
Ccam02T002266	CcWOX5	455	51.59	9.54	63.26	−0.592	Nucleus
Ccam02T002701	CcWOX6	219	24.70	8.57	57.28	−0.911	Nucleus
Ccam03T000173	CcWOX7	287	32.58	6.08	56.33	−0.959	Nucleus
Ccam03T000852	CcWOX8	276	31.42	8.26	64.5	−0.97	Nucleus
Ccam04T003014	CcWOX9	213	23.98	5.54	70.83	−0.463	Nucleus
Ccam04T003125	CcWOX10	167	19.29	7.94	61.46	−0.787	Nucleus
Ccam05T000584	CcWOX11	194	22.62	8.96	52.55	−0.831	Nucleus
Ccam07T000210	CcWOX12	524	56.83	6.51	41.86	−0.323	Nucleus and Cytoplasm
Ccam09T000192	CcWOX13	282	31.82	6.1	64.51	−0.909	Nucleus
Ccam11T000566	CcWOX14	193	22.82	8.9	61.01	−0.956	Nucleus

## Data Availability

Dataset available on request from the authors.

## Abbreviations

WOX	WUSCHEL-related homeobox
*C. camphora*	*Cinnamomum camphora*
qRT-PCR	Quantitative Reverse Transcription Polymerase Chain Reaction
BiFC	Bimolecular Fluorescence Complementation
EYFP	Enhanced Yellow Fluorescent Protein
AI	Artificial Intelligence
LBD	lateral organ boundary (LOB) domain
MYB	v-myb avian myeloblastosis viral oncogene homolog
HD	homeodomain
WUS	WUSCHEL
OC	organizing center
SAM	shoot apical meristem
CLV	CLAVATA
HMM	Hidden Markov Model
GRAVY	grand average of hydropathicity
PI	isoelectric points
TPM	transcripts per million
PH	trunk phloem
FL	flowers
FR	fruits
LE	leaves
XY	developing xylem
R	roots
ST	young stems
DAPI	4′,6-diamidino-2-phenylindole
pLDDT	Predicted local-distance difference test
CDS	coding sequences
